# Kinetic proteomics identifies targeted changes in liver metabolism and the ribo-interactome by dietary sulfur amino acid restriction

**DOI:** 10.1007/s11357-023-00758-w

**Published:** 2023-03-28

**Authors:** William O. Jonsson, Agnieszka K. Borowik, Atul Pranay, Michael T. Kinter, Emily T. Mirek, Jordan L. Levy, Elizabeth M. Snyder, Benjamin F. Miller, Tracy G. Anthony

**Affiliations:** 1 Department of Nutritional Sciences and the New Jersey Institute for Food, Nutrition and Health, Rutgers University, 59 Dudley Road – Foran Hall, Room 166, New Brunswick, NJ 08901 USA; 2 Aging and Metabolism Research Program, Oklahoma Medical Research Foundation, Oklahoma City, OK USA; 3 Department of Animal Sciences, Rutgers University, New Brunswick, NJ USA; 4 Oklahoma City VA, Oklahoma City, OK USA

**Keywords:** Healthspan, Protein synthesis, Transcriptomics, High-fat diet, Mice

## Abstract

Dietary sulfur amino acid restriction (SAAR) protects against diet-induced obesity, extends healthspan, and coincides with an overall reduction in hepatic protein synthesis. To explore the underpinnings of SAAR-induced slowed growth and its impact on liver metabolism and proteostasis, we resolved changes in hepatic mRNA and protein abundances and compared synthesis rates of individual liver proteins. To achieve this, adult male mice were provided deuterium-labeled drinking water while freely consuming either a regular-fat or high-fat diet that was SAA restricted. Livers from these mice and their respective dietary controls were used to conduct transcriptomic, proteomic, and kinetic proteomic analyses. We found that remodeling of the transcriptome by SAAR was largely agnostic to dietary fat content. Shared signatures included activation of the integrated stress response alongside alterations in metabolic processes impacting lipids, fatty acids, and amino acids. Changes to the proteome correlated poorly with the transcriptome, and yet, functional clustering of kinetic proteomic changes in the liver during SAAR revealed that the management of fatty acids and amino acids were altered to support central metabolism and redox balance. Dietary SAAR also strongly influenced the synthesis rates of ribosomal proteins and ribosome-interacting proteins regardless of dietary fat. Taken together, dietary SAAR alters the transcriptome and proteome in the liver to safely manage increased fatty acid flux and energy use and couples this with targeted changes in the ribo-interactome to support proteostasis and slowed growth.

**Supplementary Information:**

The online version contains supplementary material available at 10.1007/s11357-023-00758-w.

## Introduction

The homeostatic control of the proteome, or proteostasis, is a key determinant of metabolic health and healthy aging [1]. Proteostasis requires dynamic regulation of synthesis, folding, trafficking, and turnover processes to satisfy cellular needs against a backdrop of internal and environmental stressors and threats [2]. Disruptions in or loss of proteostasis coincides with premature aging and the development of chronic diseases and degenerative disorders [3]. Maintaining proteostasis requires a network of interacting and intersecting signal transduction pathways. One of these pathways is called the integrated stress response (ISR), which upon its activation (via phosphorylation of eukaryotic initiation factor 2, eIF2) slows protein synthesis to suppress bulk translation [4, 5]. At the same time, this signaling event triggers preferential translation of stress-responsive transcripts such as activating transcription factor 4 (ATF4) that function to realign the transcriptome towards survival and adaptation [6]. The activation of the ISR is generally considered cytoprotective, and its activation within a hormetic range may be considered salubrious [7].

Dietary restriction without malnutrition improves metabolic health and extends both lifespan and healthspan [8]. Rodents subjected to dietary sulfur amino acid (SAA) restriction (SAAR), consisting of zero cysteine plus ~80% reduction in dietary methionine by energy enjoy significant extension of lifespan, demonstrate improved glucose tolerance and are protected against high-fat diet-induced obesity (DIO) [9, 10]. In lean and DIO mice, dietary SAAR reduces synthesis rates of mixed and cytosolic but not mitochondrial protein fractions in both skeletal muscle and liver [11, 12]. Maintained protein synthesis rates in mitochondrial fractions are frequently observed in long-lived experimental models, but the molecular underpinnings remain unclear [13]. We and others observe that dietary restriction or removal of SAA triggers a non-canonical ISR, whereby the transcriptional execution of ATF4 and its target genes occurs independent of the core eIF2 phosphorylation response [11, 12, 14, 15]. Whether or not the altered transcriptome translates into similarly altered protein abundances is understudied.

Alterations in bulk fractional synthesis rates conceal the vast heterogeneity in changes in synthesis rates of individual proteins. To what extent ISR-driven changes in the hepatic transcriptome during dietary SAAR direct or reflect changes in the synthesis of individual liver proteins is unclear. Furthermore, if and how variations in dietary fat or energy density alter proteostasis outcomes during SAAR is understudied. To address these deficiencies and gaps in knowledge, we evaluated and compared changes in the transcriptome, proteome, and in the fractional synthesis rates of individual proteins during dietary SAAR in the livers of adult male mice fed either regular- or high-fat diets. Based on our earlier work, we hypothesized that the hepatic kinetic proteome would follow ISR transcriptional execution and reveal a signature of proadaptive pathways that function in metabolism and proteostasis. We observed that alterations in the transcriptome and proteome were poorly conserved when comparing an individual gene versus protein, but pathway analysis tools showed functional overlap in several biologic or metabolic processes. Key among these were alterations in the central metabolism of fatty acids and amino acids alongside changes to multiple ribosome core and interacting proteins that function in protein quality control. Importantly, these changes occurred regardless of the fat content or caloric density of the diet. These findings provide new molecular insights into how dietary SAAR alters metabolism and proteostasis in the liver.

## Materials and methods

### Animal experiments

All animal experiments were conducted in accordance with Institutional Animal Care and Use Committee (IACUC)-approved protocols at Rutgers University. To the extent possible, we have adhered to the ARRIVE guidelines 2.0 [16]. Adult male C57Bl/6J mice (between 5 and 6 months of age) were purchased from Jax and were conventionally housed in a temperature (22–24 °C) and humidity (approximately 40%) controlled AALAS-accredited facility with a 12:12 h light:dark cycle. Mice were free of all tested viruses and pathogenic agents. Mice were assigned to one of four experimental diets as previously described [11]: a regular-fat control (RF.Ctrl), RF SAAR (RF.SAAR), high-fat (HF) control (HF.Ctrl), or HF SAAR (HF.SAAR). All mice were single housed upon the start of a 7-day habituation period and were kept separate throughout the experimental periods to allow for accurate measurement of food and water consumption. All samples used in this study were derived from mice fed experimental diets for 1, 3, 7, 14, 21, or 35 days as previously described [11] and were food deprived for 4 h prior to euthanasia.

### Diets

A total of four different, isonitrogenous and cysteine-free, diets were used in this study, as previously described [11]. In both SAA-restricted diets, reductions in methionine were balanced by adding glutamic acid. Regular-fat diets were purchased from Dyets, Inc. (510071 and 510072) whereas HF diets were purchased from Research Diets, Inc. (A11051305 and A11051306). Mice had unrestricted access to food and water.

### Immunoblotting

Approximately 20 mg of frozen, crushed, liver samples were used for protein isolation. Briefly, liver tissue was homogenized in a 1:30 (weight:volume) ratio in a RIPA buffer (25 mM HEPES, 2 mM EDTA, 10 mM DTT, 50 mM sodium fluoride, 50 mM β-glycerophosphate pentahydrate, 3 mM benzamidine, 1 mM sodium orthovanadate, 0.5 % (w/v) sodium DOC, 1% (w/v) SDS, 1x protease inhibitor cocktail (P8340, Millipore-Sigma), 5 nM microcystin (33893, Millipore-Sigma)), centrifuged for 10 min at 10,000 *g* at 4 °C and supernatant collected. The protein content of the collected supernatant was measured using Pierce BCA Protein Assay (23225, Thermo Fisher Scientific). Equal volumes of supernatant and 2× sample buffer (20% (v/v) glycerol, 60 mM Tris (pH 6.8), 2% (w/v) SDS, 0.01% (w/v) bromophenol blue, 5% (v/v) β-mercaptoethanol) were mixed and heated at 95 °C for 4 min. Equal amounts of total protein were separated by denaturing polyacrylamide gel electrophoresis. Separated proteins were transferred over to PVDF membranes which were blocked for 1 h at room temperature in 5% (w/v) non-fat dry milk in TBS-T (0.1% (v/v) Tween 20). Membranes were incubated with primary antibodies (Table S1) overnight at 4 °C. After an overnight incubation, membranes were washed three times in TBS-T and then incubated with secondary antibody (Table S1) for 1 h at room temperature. After incubation with secondary antibody, the membranes were washed three times in TBS-T, after which the membranes were developed in ECL solution (RPN2235, Cytvia Amersham ECL Select Western Blotting Detection Agent, Cytvia). Membranes were imaged on an imager (ProteinSimple). Densitometry was done in AlphaView (v. 3.4.0.0, ProteinSimple).

### Hepatic mRNA abundance

Confirmatory reverse transcription quantitative PCR (RT-qPCR) analysis was performed on the same samples used in the RNA-seq. Briefly, 1 μg of RNA isolated as described above was used to generate cDNA for RT-qPCR experiments. Primers are specified in Table S2. Results were analyzed using the 2^^−ΔΔCt^ method [17], using the geometric mean of *Gapdh* and *Actb* as a reference value and expressed as fold change relative to the RF.Ctrl group.

### Serum FGF21 measurements

Concentrations of serum FGF21 were estimated as previously described [11] using a colorimetric sandwich ELISA (RD291108200R, BioVendor) according to the manufacturer’s instructions. Serum samples were diluted 10 times prior to analysis, with the resulting absorbance measured spectrophotometrically (SpectraMax M2, Molecular Devices).

### Liver NAD^+^/NADH measurements

Hepatic ratios of NAD^+^/NADH ratios were estimated using a colorimetric assay (MAK037, Sigma-Aldrich) according to the manufacturer’s instructions, with the following notes on the procedure. Approximately 20 mg of frozen, crushed, liver samples were used for ratio estimations and homogenized in the provided extraction buffer on ice using disposable polypropylene pestles. Samples were deproteinated using 10 kDa molecular weight cutoff spin columns by centrifuging cleared lysates for 5 min at 14,000 × *g*, at 4 °C. Filtrates were diluted 1:1 in extraction buffer and split into two aliquots, one for total NAD^+^ and one for NADH determination, as per the manufacturer’s instructions, with final volumes of 25 μL. Samples were analyzed in duplicates by loading 4–5 μL (kept consistent within individual assays, i.e., day 7 and day 35 measurements) of the above samples and diluting reactions further as per the manufacturer’s instructions and incubating reactions for 2 h at room temperature followed by measuring absorbance (at 450 nm) on a spectrophotometer (SpectraMax M2, Molecular Devices).

### Kinetic proteomics sample preparation

Liver samples from mice (*n* = 3/group/timepoint) euthanized after 1, 3, 7, and 14 days of feeding experimental diets were used for protein isolation for subsequent kinetic proteomics. Animals were deuterium labeled as previously described [18]. Briefly, all mice received an initial i.p. bolus injection of 99% D_2_O equivalent to 5% of the body water pool followed by 8% D_2_O-enriched drinking water for the designated labeling period. Approximately 20 mg of the frozen powdered liver was lysed in 1 mL modified RIPA buffer (0.5% (w/v) SDS, 0.1% (v/v) Triton X-100, 11 mM sodium DOC, 1 mM EDTA in TBS, pH 7.6) by three cycles of freezing in liquid N_2_ and thawing at 37 °C, heated at 70 °C for 15 min, and centrifuged for 5 min at 500 *g* at 4 °C after which the supernatant was collected to be used for further downstream analysis. The protein content of the collected supernatant was measured using Pierce BCA Protein Assay (23225, Thermo Fisher Scientific) and used for subsequent equilibration as detailed below.

### Body water enrichment

To determine body water enrichment, 50 μL of serum was placed into the inner well of o-ring screw cap and inverted on an 80 °C heating block overnight. Distilled samples were diluted 1:300 in ddH_2_O and analyzed on a liquid water isotope analyzer (Los Gatos Research, Los Gatos, CA, USA) against a standard curve prepared with samples containing wide range concentrations of D_2_O [18].

### Kinetic proteomics and analysis of isotope incorporation

Protein samples were prepared as previously described [19, 20]. Briefly, 80 μg of total protein was taken for analysis, and 8 pmol BSA was added to the protein samples in 1% SDS as an internal standard. The total protein was desalted by precipitation in 1 mL of acetone overnight at −20 °C. The protein pellet was solubilized in 50 μL Laemmli sample buffer, and 20 μg protein was run in a 12.5% SDS-Page gel (BioRad Criterion system). The gels were fixed and stained with Coomassie blue (GelCode blue, Pierce Chemical Company). Each sample was cut from the gel as the entire lane and divided into smaller pieces. A standard in-gel digestion method was used [20]. The gel pieces were washed to remove the Coomassie blue and then reduced in 10 mg/mL DTT, alkylated in 35 mg/mL iodoacetamide, and digested overnight with 1 μg trypsin per sample in 200 μL 10 mM ammonium bicarbonate. The mixture of peptides was extracted from the gel, evaporated to dryness in a SpeedVac, and reconstituted in 150 μL 1% acetic acid (v/v) for liquid chromatography (LC)-tandem mass spectrometry (MS) analysis.

Protein concentration and isotopic distribution were evaluated by LC-high resolution MS. We used a QEx Plus hybrid quadrupole-orbitrap mass spectrometry system (ThermoScientific), a splitless nanoflow HPLC system with autoinjector (ThermoScientific), and a 10-cm C18 column (Phenomenex Aeris 3.6 μm Peptide XB-C18 100A) packed in a fused silica electrospray tip (New Objective). Five microliter sample volumes were injected and loaded onto the column at 1.5 μL/min with 0.1% formic acid. The column was eluted at 150 nL/min with a linear gradient of CH3CN in water with 0.1% formic acid (2% CH3CN to 65% CH3CN in 60 min). The orbitrap mass spectrometer acquired full scan mass spectra with a m/z resolution of 280,000. Ion source settings included a spray voltage of 2.0 kV, ion transfer tube temperature of 300 °C, and positive ion mode. The high-resolution accurate mass (HRAM) analyses were managed through the program Skyline (MacCoss laboratory) [21] and included at least two unique peptides from each protein. The proper detection of all peptides used for these analyses was validated during the method development process.

### Identification of peptides/proteins

We used Mascot Daemon (version 2.8.0) from Matrix Science for a base search of tandem mass spectra to identify peptides/proteins. The following parameters were used for mascot searches: precursor mass accuracy set to 15 ppm, fragment ion mass accuracy set to 0.6 Da, carbamido-methylation of Cys was the fixed modification, and oxidation of Met and acetylation of Lys were set as dynamic modifications. Trypsin specificity of peptides was used, and up to 2 missed cleavages were allowed. The SwissProt database and (*Mus musculus*) mouse taxonomy were both used. The false discovery rate (FDR) of peptide-spectrum match was controlled by using the decoy database approach. The FDR cutoff was set at 5% (0.05).

### Protein synthesis rate calculations

Protein synthesis rates were determined using d2ome software [22], which allows for automated quantification of isotopomers of tryptic peptides (both endogenous mass and heavier deuterium-enriched species). Using d2ome, the protein synthesis rates were determined using the time course of deuterium incorporation and a nonlinear regression fit model. D2ome makes these calculations by determining the rate of decline of the M0 isotopomer as the mass shifts from the M0 isotopomer to heavier mass isotopomers (e.g., M+1, M+2, M+3) with the incorporation of deuterium over time. The individual protein synthesis rates were calculated using the median value of all peptides for a protein. To minimize the impact of variability within quantifying peptides, Grubbs̕ outlier detection and removal was used.

### Gene expression profiling

Liver samples from mice (*n* = 3/group) euthanized after 7 days of feeding experimental diets were used for RNA isolation for subsequent gene expression profiling (RNA-seq). Isolation of RNA was performed by adding approximately 20 mg of crushed and frozen liver samples to TRI Reagent RT (RT 111, Molecular Research Center, Inc.) and use of Direct-zol RNA Miniprep (R2052, Zymo Research) according to the recommended protocol. The sufficient quantity and quality of the isolated RNA were confirmed using NanoDrop One (Thermo Fisher Scientific) and Bioanalyzer 2100 (Agilent Technologies, Inc.). External quality control of the isolated RNA, polyA selection, library preparation, and 150PE Illumina sequencing was performed by GENEWIZ (GENEWIZ, South Plainfield, NJ, USA). The raw reads were processed as follows: initial quality control of FASTQ data using FastQC (version 0.11.9) and adapter and quality trimming using cutadapt (version 3.4) [23]. Pseudoalignment was performed using kallisto (version 0.46.2) [24]. Mouse Ensembl Transcriptome v94 was the reference genome. Differential gene expression analysis was performed by loading kallisto output into R (version 4.0.3, https://www.r-project.org/) running RStudio (https://posit.co/) and using tximport (version 1.18.0) [25] followed by running DEseq2 (version 1.30.1) with independent filtering [26]. Differentially expressed genes (DEGs) were defined by an adjusted *p*-value (padj) of <0.05 without any constraints on the level of fold change (log2FC). Gene ontology (GO) analysis was performed as described under pathway analysis, analyzing up (log2FC > 0) and down (log2FC < 0) DEGs separately. Additional information and the raw and processed data are available on the Gene Expression Omnibus repository under identifier GSE200149.

### Pathway analysis for gene expression data

Gene set enrichment analysis (GSEA) [27] was used to identify pathway enrichments related to down and upregulated DEGs with padj <0.05. For the GSEA, we manually selected the following GSEA collections for a more focused enrichment analysis: C1, C3, CP, GO, and H. The top 10 up and down pathways, by enrichment adjusted *p*-value, for select comparisons are displayed, including pathway member abundance (k/K) information.

### Pathway analysis for kinetic proteomics data

Pathway analysis was performed using the STRING database (version 11.5) [28], with evidence stringency set to 0.7 (“high”) for the initial pathway enrichment analysis (Fig. 5) and then maintained at 0.4 (“medium”) for subsequent analyses. Pathway analysis was limited to those proteins that were reliably detected in both groups of comparison with 562 serving as the minimum number.

### Data visualization and statistical analysis

All analyses and visualization of data were conducted in R (version 4.0.3) running RStudio (https://posit.co/) using the following packages: tidyverse [29], UpSetR [30], car, rstatix, ggpubr, and DescTools. Figures and illustrations were compiled in Adobe Illustrator (version 25.4.1, Adobe). Adherence to statistical test assumptions (normality and homoscedasticity) was assessed by using the Shapiro-Wilk and Levene’s tests. When necessary, data was log-transformed to approach adherence. Statistical main or interaction effects between independent variables were analyzed using either two- or three-factor analysis of variance (2- or 3-way ANOVA) with either diet (Ctrl or SAAR) and fat (RF or HF) content as main variables in the case of 2-way ANOVA and with diet, fat, and time in the case of 3-way ANOVA. Comparisons between groups were conducted using pairwise *t*-tests with either Bonferroni or FDR correction for multiple comparisons. Wherever relevant, data is presented as mean ± standard error of the mean (sem) with individual data points displayed as dots. Statistical significance, unless otherwise specified, was set to *α* = 0.05.

### Data availability

The data supporting the findings in this article are either available through data deposited at online repositories (Proteomics: 10.5281/zenodo.7215980, Transcriptomics: GSE200149) or available from the corresponding author upon reasonable request.

## Results

### Dietary SAAR promotes leanness and triggers amino acid stress signaling events in the liver independent of dietary fat content and energy consumption

**Fig. 1:**
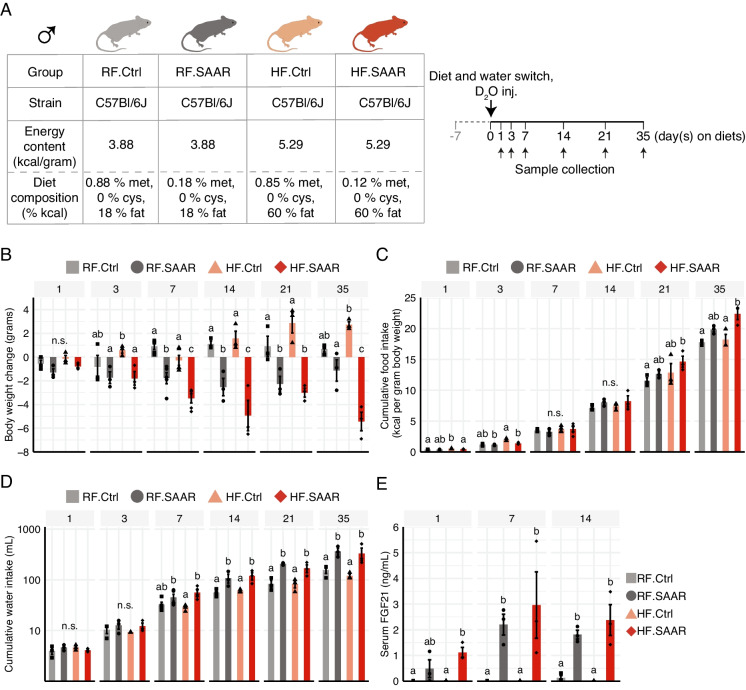
Dietary sulfur amino acid restriction triggers hyperphagia, hyperdipsia, and attenuation of weight gain independent of dietary fat and energy content. **A** Study outline with information on biological sex, group names, mouse strain, dietary energy content, sulfur amino acid content, and key experimental interventions, including sample collection (small arrows) and intraperitoneal administration of deuterium (D_2_O inj.) and commencement of experimental diets and deuterium in drinking water (large arrow). The four diet groups are regular-fat control diet (RF.Ctrl, light grey square), regular-fat sulfur amino acid restricted diet (RF.SAAR, dark grey circle), high-fat control diet (HF.Ctrl, peach triangle), and high-fat sulfur amino acid restricted diet (HF.SAAR, red diamond). **B** Change in body weight of animals euthanized at the timepoints indicated. **C** Cumulative food intake normalized to body weight. **D** Cumulative water intake of animals euthanized at indicated timepoints. **E** Serum levels of fibroblast growth factor 21 (FGF21) measured in mice euthanized after 1, 7, or 14 days of consuming experimental diets. Data is displayed as mean ± standard error of the mean, with individual data points displayed as dots (*n* = 3–5 male mice per group). Within each timepoint (indicated in grey headers), bars without shared letters were statistically different at *α* = 0.05, as determined by 3-way ANOVA followed by pair-wise *t*-tests with (**B**, **C**, and **D**) FDR or **E** Bonferroni’s correction for multiple comparisons

In order to confirm previous findings that dietary SAAR defends against DIO, we fed adult male C57Bl/6J mice either regular-fat (18% of energy) or high-fat (60% of energy) SAAR diets for up to 35 days (Fig. 1A). Mice fed either SAAR diet lost weight, especially towards the end of the dietary intervention, whereas mice fed either regular-fat or high-fat control diets gained weight throughout the study period (Fig. 1B and Figure S1A). Furthermore, mice consuming a HF.SAAR diet lost the most body weight even though they consumed the most calories (Fig. 1B, C and Figure S1B-C). In addition, both cumulative and estimated daily water consumption increased during dietary SAAR (Fig. 1D and Figure S1E). Increased daily water and food consumption were established by days 7 and 14, respectively, in both RF.SAAR and HF.SAAR mice, and these measurements were associated with circulating concentrations of FGF21 (Fig. 1E and Figure S1D and S1F).

**Fig. 2:**
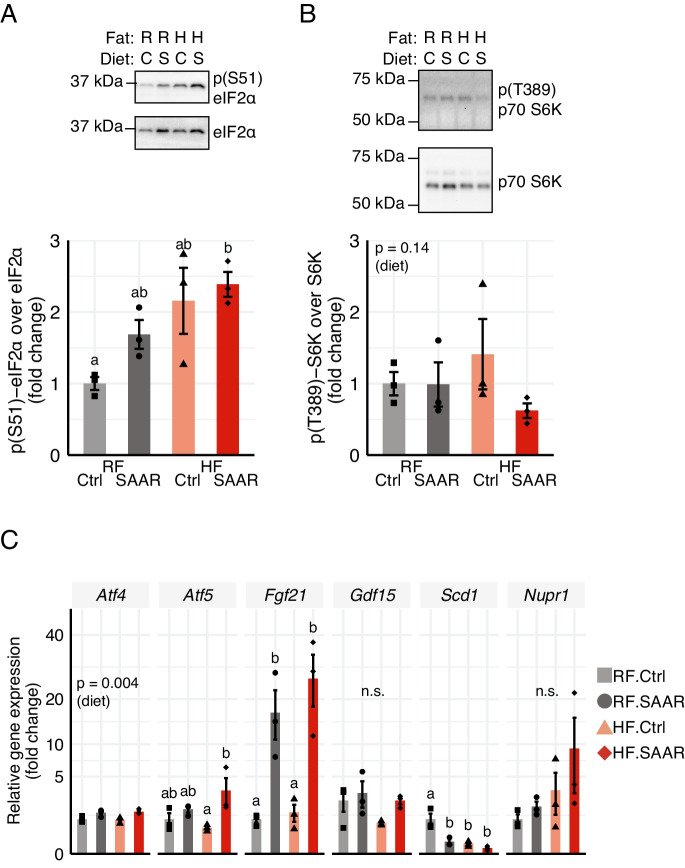
Seven days of dietary sulfur amino acid restriction activates the integrated stress response in the livers of male mice. **A** Hepatic ratios and representative blots of **A** phosphorylated (at S51) eIF2α over total eIF2α and **B** phosphorylated (at T389) p70 S6K over total p70 S6K in mice fed a regular-fat control (RF.Ctrl, light grey bars), RF sulfur amino acid restricted (RF.SAAR, dark grey bars), high-fat control (HF.Ctrl, peach bars), or HF.SAAR (red bars) for 7 days. **C** Hepatic ratios of (from left to right) *Atf4*, *Atf5*, *Fgf21*, *Gdf15*, *Scd1*, and *Nupr1* in mice fed indicated diets for 7 days. Data is displayed as mean ± standard error of the mean, with individual data points displayed as dots (*n* = 3–5 mice per group). Bars without shared letters were statistically different at *α* = 0.05, as determined by 2-way ANOVA followed by pair-wise *t*-tests with Bonferroni’s correction for multiple comparisons

Increased hepatic phosphorylation of eIF2 on its alpha subunit, indicative of ISR activation, was evident at day 7 in both SAAR and HF groups, reaching the highest levels in the HF.SAAR group (Fig. 2A). Another sensor and transducer of amino acid status is the mechanistic target of rapamycin complex 1 (mTORC1). Hepatic mTORC1 signaling was unchanged by diet at day 7 (Fig. 2B) but became suppressed by SAAR over time. By day 35, both ISR activation and mTORC1 suppression were evident in the livers of SAAR-fed mice, independent of dietary fat and GCN2 phosphorylation (Figure S2A-D). ISR activation was further confirmed in the livers of SAAR-fed mice at day 7 via an increased abundance of several ATF4 target genes, including *Atf4*, *Atf5*, *Fgf21*, and decreased *Scd1* expression (Fig. 2C). However, no alterations in the ATF4 gene targets *Gdf15* and *Nupr1* were noted (Fig. 2C). Collectively, dietary SAAR induced robust and sustained phenotypical changes alongside molecular markers of ISR activation and diminished mTORC1 signaling regardless of dietary fat content.

### Dietary SAAR is agnostic to dietary fat content in reshaping the liver transcriptome

**Fig. 3:**
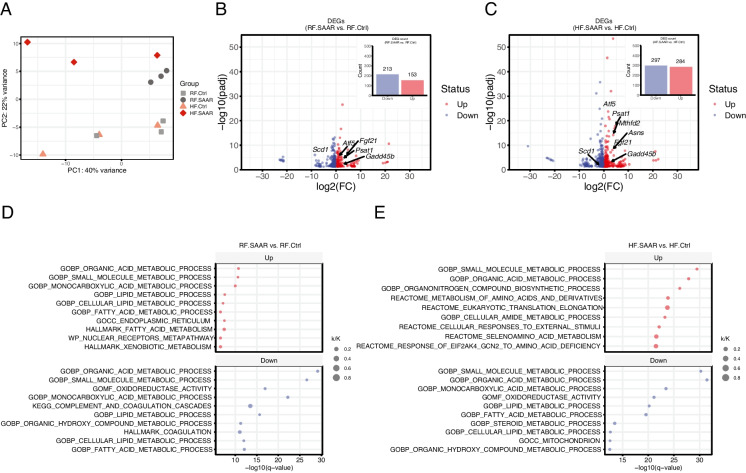
Seven days of dietary sulfur amino acid restriction realigns the hepatic transcriptome to favor increased fatty acid metabolism independent of dietary fat content. **A** Principal component analysis (PCA) plot for individual liver samples from mice fed either a regular-fat control (RF.Ctrl, light grey squares), RF sulfur amino acid restricted (RF.SAAR, dark grey circles), high-fat control (HF.Ctrl, peach triangles), or high-fat sulfur amino acid restricted (HF.SAAR, red diamond) diets for 7 days. Data based on RNA-seq data showing separation by principal component (PC) 1 and PC2. **B** and **C** Differentially expressed gene (DEG, defined as *q* <0.05)-related volcano plot highlighting up- and down-regulated transcripts and total counts of each (inset bar graph) for **B** RF.SAAR vs. RF.Ctrl and **C** HF.SAAR vs. HF.Ctrl, with integrated stress response (ISR) target transcripts labeled. **D** and **E** Top 10 up- and down-regulated pathways based on gene set enrichment analysis of DEGs in **D** RF.SAAR vs. RF.Ctrl and **E** HF.SAAR vs. HF.Ctrl. Pathway member abundance (k/K) is indicated by shape size and enrichment significance is indicated on the *x*-axis. *n* = 3 male mice per group

We performed RNA sequencing on liver samples collected 7 days after the start of experimental diets (Table S3). Principal component analysis (PCA) showed a clear separation between liver samples from Ctrl- and SAAR-fed mice (Fig. 3A). Of note, PCA did not indicate clear separation by dietary fat content. Rather, HF feeding increased sample heterogeneity. We then searched the differentially expressed gene lists for ISR-gene targets as identified in previously published lists [31, 32] (Table S4). We noted in both SAAR groups increased expression of *Fgf21*, *Atf5*, and other ISR-related transcripts*,* as well as reduced expression of *Scd1*, when compared to their respective controls (Fig. 3B, C). Indeed, the liver ISR gene signature in mice fed HF.SAAR was augmented versus mice fed RF.SAAR, evidenced by Reactome pathways “eukaryotic translation elongation” and “response to eIF2AK4/GCN2 to amino acid deficiency” present only in the high-fat comparison (Fig. 3D, E). The amplified ISR signature may be due to the protein dilution effect of high-fat feeding because mice fed HF.SAAR consumed less protein and SAA on a weight basis than mice fed RF.SAA (Figure S1B).

Gene set enrichment analysis (GSEA) on DEGs indicated that both RF.SAAR and HF.SAAR increased the expression of fatty acid metabolic genes relative to their respective control groups (Fig. 3 D, E). When comparing the two SAAR groups, GSEA corroborated the observed phenotype of body weight reduction by highlighting greater differences in the expression of genes related to fatty acid and triglyceride metabolism, such as *Scd1* (Figure S3A-B). While the response amplitude of several genes was greater in HF.SAAR, the gene lists themselves were quite similar. We interpret these results to indicate that dietary SAAR alters the hepatic transcriptome to favor increased fatty acid metabolism regardless of the dietary fat content.

Another hallmark of ISR activation and SAAR is altered redox status and improved redox defenses. In this regard, we noted that the GO molecular function term “oxidoreductase activity” was found among the top 10 down pathways in both SAAR vs. Ctrl comparisons (Fig. 3 D, E). Given the central role that redox status plays in the maintenance of mitochondrial health and healthy aging, we further investigated this term and the associated DEGs, revealing a relatively large overlap between both comparisons (Figure S3C). Among the overlapping downregulated transcripts were multiple genes encoding dehydrogenases and mitochondrial-encoded proteins, pointing to processes involved with the consumption of cellular reducing power by the mitochondria.

### Dietary SAAR changes the composition of proteins in the liver that make up and interact with the ribosome

**Fig. 4:**
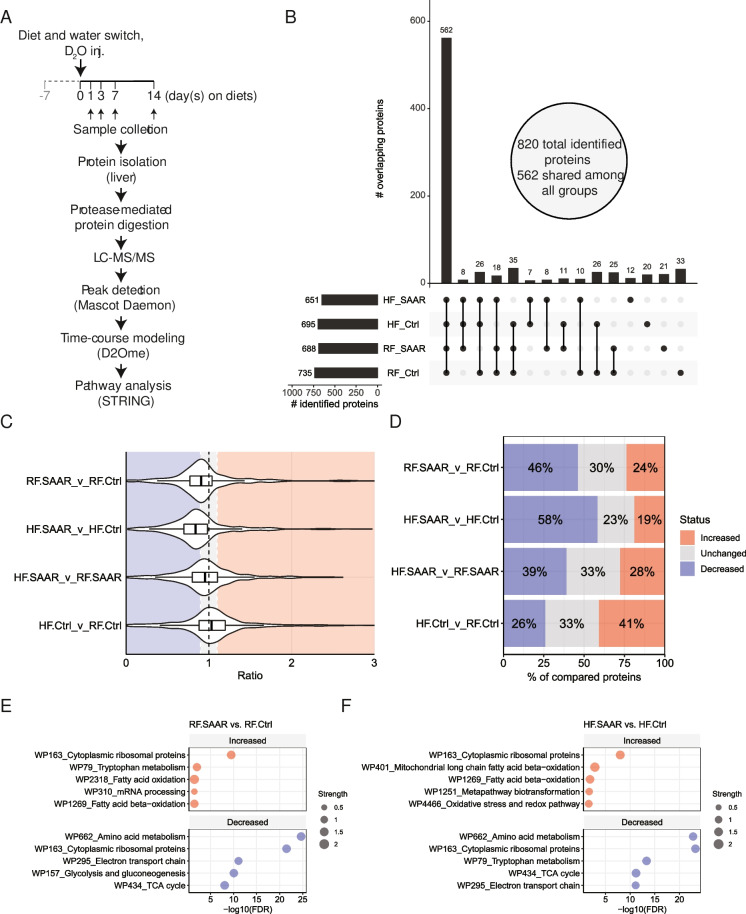
Dietary sulfur amino acid restriction alters synthesis rates of specific hepatic proteins to accommodate increased fatty acid metabolism. **A** Overview of kinetic proteomic approach. Small arrows indicate when (1, 3 7, or 14 days after the start of experimental diets) liver samples were collected for subsequent kinetic proteomics. **B** Upset plot displaying the number of proteins, of 820 total identified proteins, found overlapping between groups, as indicated by vertically connected dots along the *x*-axis. Dots without vertical connections indicate cases when proteins were found only in a single group: regular-fat control (RF.Ctrl), RF sulfur amino acid restricted (RF.SAAR), high-fat (HF) control (HF.Ctrl), or HF.SAAR. Total protein counts in each group are indicated by horizontal bar graphs. **C** Distributions of synthesis rate ratios in the experimental groups: regular-fat control (RF.Ctrl), RF sulfur amino acid restricted (RF.SAAR), high-fat Ctrl (HF.Ctrl), or HF SAAR (HF.SAAR)) comparisons. **D** Percentage of compared proteins defined as either increased (>1.1 ratio), decreased (<0.9 ratio), or unchanged in their synthesis rates in the respective comparisons. **E** Top 5 WikiPathway (WP) terms for proteins with either increased (top) or decreased (bottom) synthesis rate ratios in RF.SAAR vs. RF.Ctrl and **F** Top 5 WikiPathway (WP) terms for proteins with either increased (top) or decreased (bottom) synthesis rate ratios in HF.SAAR vs. HF.Ctrl. Enrichment strength is indicated by shape size and false discovery rate (FDR) is displayed on the *x*-axis. Data is a composite of *n* = 3 male mice per group per timepoint

Knowing that dietary SAAR reduces bulk protein synthesis in the liver [11, 12], we turned our attention to the hepatic proteome and examined how synthesis rates of individual proteins were altered. Employing a kinetic proteomics approach (Fig. 4A), 820 unique liver proteins were identified across the four different experimental groups. Of these, 562 proteins were present in all four groups (Fig. 4B), thus allowing for a rich investigation of individual protein synthesis rate changes between and among groups (Table S5). Proteins identified only in a single group (Fig. 4B, the last four bars from the left and Table S6) did not represent any specific biological processes when submitting to pathway analysis and therefore were not included in subsequent pairwise comparisons.

To better understand relative differences between groups, a magnitude-based inference approach was used to analyze differences in synthesis rate ratios with each pairwise comparison [33]. Using this approach, we decided a 10% or greater change would represent a physiologically or practically important value of effect. This value is based on our previous analysis of liver protein synthesis using null-hypothesis significance testing which showed that SAAR reduced liver protein synthesis by 10% or more [11, 12]. As such, we defined three response categories in pair-wise comparisons of interest: we defined synthesis rate ratios <0.9 as “decreased,” >1.1 as “increased,” and ratios between 0.9 and 1.1 as “unchanged.” Consistent with our published findings that dietary SAAR reduces bulk protein synthesis in the liver, we observed an overall left shift, or reduction, in synthesis rate ratios for both SAAR diets as compared to their respective control diets. This was noted whether the data were visualized as a violin plot to show the density of distributed data (Fig. 4C) or more simply as response category percentages (Fig. 4D). We also noted that high-fat feeding resulted in a rightward shift, or increase, in ratio distribution as compared to feeding the low-fat diet.

**Fig. 5:**
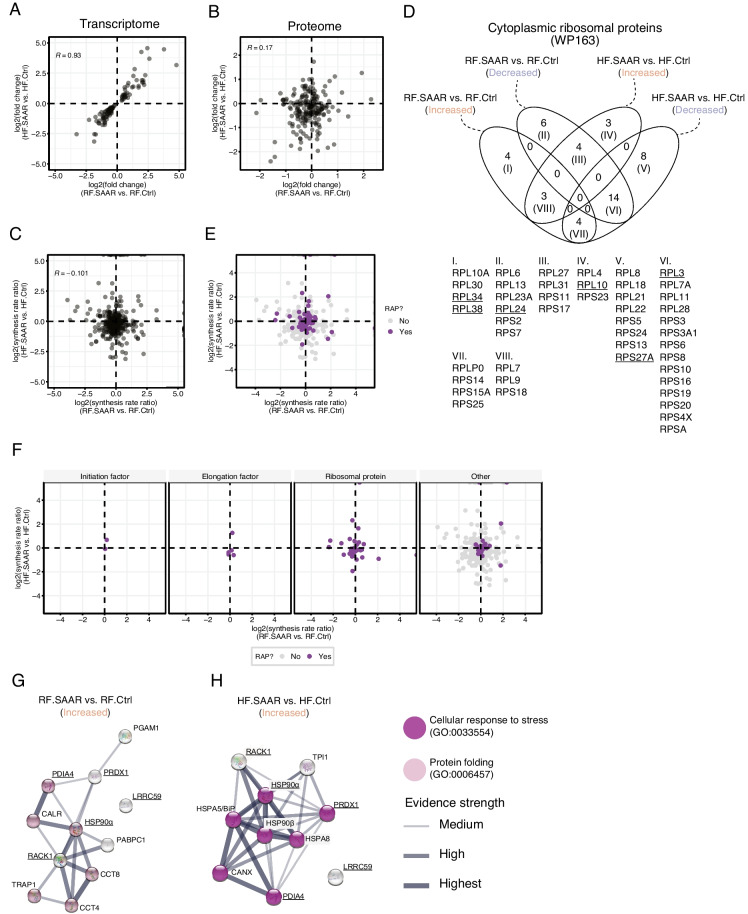
Dietary sulfur amino acid restriction alters protein synthesis rates of ribosomal proteins and ribosome-associated proteins in the liver. **A** Transcriptomic correlation between differentially expressed genes (DEGs) in the regular-fat sulfur amino acid restriction (RF.SAAR) vs. RF control (RF.Ctrl) and the high-fat SAAR (HF.SAAR) vs. HF.Ctrl comparisons. **B** Proteomic correlation between relative changes in hepatic protein expression at day 7 in the RF.SAAR vs. RF.Ctrl and the HF.SAAR vs. HF.Ctrl comparisons. **C** Correlation between protein synthesis rate ratios in the RF.SAAR vs. RF.Ctrl and the HF.SAAR vs. HF.Ctrl comparisons. Pearson’s correlation coefficient (Pearson’s *r* or *R*) is displayed for each correlation using the plotted data. **D** Venn diagram showing the classification (increased or decreased in synthesis rate ratio) of the ribosomal proteins comprising the WikiPathway (WP) term “cytosolic ribosomal proteins.” Roman numerals within the Venn diagram refer to the below table showing which ribosomal proteins were found in a specific comparison. Underscored ribosomal proteins signify ones described to have a rapid turnover within the assembled 80S ribosome, as detailed in the main text. **E** Correlation between synthesis rate ratios of ribosome-associated proteins (RAPs) in the RF.SAAR vs. RF.Ctrl and the HF.SAAR vs. HF.Ctrl comparisons. Purple dots highlight identified RAPs. **F** Separated correlations (from **E**) of different classes of RAPs (left to right: initiation factors, elongation factors, ribosomal proteins, and others) in the RF.SAAR vs. RF.Ctrl and the HF.SAAR vs. HF.Ctrl comparisons. **G** and **H** pathway enrichment analysis network of RAPs within the “others” category with increased synthesis rate ratios in the **G** RF.SAAR vs. RF.Ctrl and **H** HF.SAAR vs. HF.Ctrl comparisons. Legend for **G** and **H** indicate enriched gene ontology (GO) terms and evidence strength as defined by STRING. Data is a composite of *n* = 3 male mice per group per timepoint

Next, commonalities among proteins with increased or decreased rate ratios were explored using pathway enrichment analysis. We observed substantial overlap among the top 5 increased and decreased WikiPathway (WP) terms (Fig. 4C, D; Figure S4C-D; and Table S7). A major signature shared in both SAAR groups was the term “Cytoplasmic ribosomal proteins” (WP163) (Fig. 4C, D and Table S7). To explore this further, we analyzed relative hepatic protein abundances at day 7 (Table S8). Correlating the fold changes in protein abundance between RF.SAAR vs. RF.Ctrl and HF.SAAR vs. HF.Ctrl comparisons, we noted that the common differential response to SAAR at the proteome level was relatively weak (Pearson’s *r* = 0.17) as compared to the transcriptome (Pearson’s *r* = 0.93, using only DEGs) (Fig. 5A, B). A weak shared differential response to SAAR was also observable at the level of protein synthesis rates (Fig. 5C), showing a limited correlation of synthesis rate ratios (Pearson’s *r* = −0.101) between the two dietary fat comparisons.

To explore the role of the translational machinery itself in this, we processed the ribosomal proteins (RPs) that comprised the WP-term “Cytoplasmic ribosomal proteins” (WP163) and identified in which category they fell (“increased” or “decreased,” as defined above) and noted a complex relationship (Fig. 5D). While a large collection of RPs had decreased synthesis rate ratios in either one or both comparisons (Fig. 5D, tulip areas II, V, and VI), there were several that showed discordant changes (Fig. 5D, tulip areas III and VII).

In light of recent data on differential exchange rates among the core RPs into assembled ribosomes, we used previously established classifications of RPs under dietary restriction conditions [34] as either (a) static, the category in which approximately 80% of core RPs fall into; (b) rapidly exchanging, the category of core RPs that can be replaced during the lifetime of a ribosome; or (c) conditionally either static or rapidly exchanging. Among the RPs that had been classified as rapidly exchanging under dietary restriction (RPL3, RPL10, RPL19, RPL24, RPL38, RPS27A), we noted that a majority were uniquely represented in only one comparison, with one-half increasing (RPL10, RPL30, and RPL34) and the other half decreasing (RPL3, RPL24, and RPS27A) in synthesis rate ratios (Fig. 5D). We noted limited differential gene expression of identified RP transcripts in our transcriptomics data (Figure S5).

Ribosome heterogeneity is suggested to extend beyond the core RPs to include other proteins that directly interact with the ribosome, termed ribosome-associated proteins (RAPs), that together with core translational proteins form the ribo-interactome [35]. By filtering our synthesis rate dataset through the published ribo-interactome in mouse embryonic stem cells, we identify 89 RAPs with different synthesis rate ratios in SAAR vs. Ctrl comparisons (Fig. 5E and Table S9).

Upon further manual classification of these RAPs, we first noted that the few identified translation initiation (eIF4A1 and eIF5A) and elongation (eEF1A1, eEF1B, eEF1D, eEF1G, eEF2) factors changed very little in synthesis rate whereas many RPs and other RAPs not included in the core translational machinery displayed greater changes (Fig. 5F). Pathway enrichment analysis identified several overlapping RAPs between the two comparisons, namely RACK1, LRRC59, PRDX1, and HSP90α, plus PDIA4. These proteins clustered into the GO biological process (BP) “Protein folding” (GO:0006457) in the RF comparison (Fig. 5G) whereas the GO BP term “Cellular response to stress” (GO:0033554) emerged in the HF comparison (Fig. 5H). Taken together, these data suggest that core RP and RAP stoichiometry undergo rearrangement during SAAR to improve protein quality control.

### Dietary SAAR reshapes the proteome to alter amino acid and fatty acid metabolic processes in the liver

Both SAAR groups when compared to their respective controls had the WP terms “amino acid metabolism” (WP662), “TCA cycle” (WP434), and “electron transport chain” (WP295) as the top terms for proteins with reduced synthesis rate ratios. With respect to “amino acid metabolism” (WP662), several methionine metabolism-related proteins (Table S7) were represented, including cystathionine gamma-lyase (CGL), cystathionine beta-synthase (CBS), mercaptopyruvate sulfurtransferase (MPST), and glutathione S-tranferase A4 (GSTA4), likely indicative of reduced synthesis rates of proteins involved in the downstream metabolism of both cysteine and methionine. Also among the most decreased pathways in the HF.SAAR vs. HF.Ctrl comparison was the WP term, “tryptophan metabolism” (WP79) consisting of proteins involved in the de novo biosynthesis of NAD^+^ alongside several dehydrogenases (e.g., ALDH1A1, ALDH2, ALDH3A2, HADH, and HSD17B10) which function in NAD^+^/NADH use (Figure S6A-C). Paradoxically, this term was also among the most increased pathways in the RF.SAAR vs. RF.Ctrl comparison (Fig. 4C, D), and all identified proteins in the RF.SAAR vs. RF.Ctrl comparison were present in the HF.SAAR vs. HF.Ctrl comparison. Analysis of hepatic ratios of NAD^+^/NADH in samples collected both after 7 and 35 days of experimental diets did not reveal any statistically significant differences among groups (Figure S6D-E). Hepatic expression of two major NAD^+^-consuming proteins (SIRT1 and PARP1) also did not differ at day 35 (Figure S6F-G). While the synthesis rates for several proteins involved in AA usage were altered so as to suggest AA stress, none of the altered proteins were products of known ISR target genes (e.g., those illustrated in Fig. 3 or listed in Table S4).

Finally, we noted that dietary SAAR increased synthesis rates of proteins related to fatty acid oxidation in the liver regardless of dietary fat level. Both SAAR vs. Ctrl comparisons revealed an enrichment for increased synthesis of proteins involved in fatty acid oxidation (WP2318, WP1269, and WP401) (Fig. 4C, D). These lists (Table S7) included proteins involved in peroxisomal fatty acid oxidation (EHHADH and PECR), mitochondrial fatty acid oxidation (ACADM, ACADS, ACADVL, ACSL1, ECHS1, GCDH, and HADHB), and other facilitating processes (TPI1 and MTTP) that are consistent with the physiological condition of losing body fat. Livers from HF.SAAR mice also showed the increased synthesis of proteins involved in neutralizing ROS and maintaining cellular redox homeostasis (WP1251, WP4466). In summary, kinetic proteomic changes in metabolism reflected mobilization, catabolism, and utilization of fatty acids alongside ROS management and amino acid sparing as seen during an adaptive starvation response.

## Discussion

Understanding how dietary restriction strategies modulate proteostasis is a crucial step in the development of healthspan-extending dietary treatments [8, 36, 37]. Here, we build on previous data in the liver by identifying distinct processes in metabolism and proteostasis that were altered by dietary SAAR in male mice. These changes were characterized by an increase in the synthesis of fatty acid oxidation proteins, a decrease in the synthesis of amino acid metabolic proteins, and a remodeling of proteins involved in ROS handling and NAD+ metabolism. Importantly, we find that the proteostatic machinery itself is impacted, with the synthesis of many RPs and RAPs significantly altered in the liver by dietary SAAR. Collectively, these changes are compatible with a slowed growth phenotype coupled with improved cellular maintenance and stress resilience. This work contributes to research efforts in healthy aging by providing a rich dataset representing an adult baseline in a species widely studied to understand the mechanism by which dietary SAAR improves healthspan and extends lifespan [9].

In addition to confirming ISR activation to dietary SAAR, we observed a clear transcriptomic response that was largely agnostic to the fat and energy content of the diet. A slightly amplified ISR signature in the HF.SAAR group is likely the consequence of a greater protein dilution effect [38]. The protein and SAA content of diets used in this study were designed to be similar on a per-weight basis. Mice consuming a HFD consumed more calories but less absolute intake in grams, further reducing the absolute intake of all essential AAs in addition to the sulfur amino acids. Thus, a greater degree of restriction elicited a greater response. This finding emphasizes how the liver is exquisitely responsive to protein quality and quantity, regardless of energy intake.

In stark contrast to the transcriptomic data, we were unable to detect a single hallmark ISR protein from our manually curated list in our kinetic proteomic dataset. Certainly, we and others have observed an increased abundance of ISR-associated proteins and target transcripts upon SAA restriction or deprivation using other methods of detection [11, 12, 14, 15, 39–42]. This difference between ISR transcript versus protein levels may be consequential to ISR gene targets possessing a relatively short protein half-life (i.e., minutes to hours) or existing in low abundance. Calculation of protein synthesis using deuterium-labeled alanine is resolved best in relatively abundant proteins across days to weeks, and we previously reported *Atf4* translation occurs within minutes upon feeding a diet deficient in SAA [14].

Another important observation in our study is that RP synthesis rates are dynamically regulated during SAAR. In budding yeast, methionine restriction reduces ribosomal gene abundances and alters ribosome loading patterns in the 5′ untranslated regions of genes, affecting translation efficiency [43]. Similarly, targeted screens of RP mRNA translation during acute conditions of nutrient stress in cells and in the liver shows that RP-encoding transcripts are preferentially suppressed translationally via mTORC1 and the ISR [44–47]. There is a very limited amount of data on RP translation during dietary SAAR. Our current results suggest that translational control during SAAR is more complex and varied than previously appreciated. Recent studies in eukaryotic cells identify distinct heterogeneous RP stoichiometry during stress [48, 49]. Peripheral RPs such as RPL38 are replaced multiple times during the lifespan of a ribosome and are sensitive to dietary signals [34]. Ribosomal proteins such as RPL38 also participate in regulating the translation of a subset of genes via interaction with structured RNA elements [50]. Data such as these point to an additional layer of ribosome-mediated translational control in which heterogeneity of surface RPs may influence subsets of transcripts during dietary SAAR.

Ribosomes associate with a large pool of accessory proteins; ~430 of these proteins interact directly with ribosomes and are defined as ribosome-associated proteins or RAPs [35]. Recent work shows that RAPs can interact with specific subsets of ribosomes to regulate translation at defined subcellular locations [35]. One example of such an increased RAP is the peroxidase, peroxiredoxin 1 (PRDX1). Peroxiredoxins function as protective proteins and help to maintain cellular redox status under nutrient-stress conditions [51]. An increase in the synthesis of RAPs such as PRDX1 may serve as a means of hepatocellular protection during dietary SAAR.

We also note that other pathways related to redox balance, exemplified by tryptophan metabolism and its relationship with NAD^+^ biosynthesis, had differential regulation of synthesis rates and as a function of dietary fat content. Nonetheless, we did not detect differences in whole liver lysate NAD^+^/NADH ratios. We interpret these results to suggest that SAAR-driven and fat-dependent protein synthesis changes are consequential to a higher priority to maintain NAD^+^/NADH flux, as previously characterized in calorically restricted and aged mice [52]. Given the central role that redox balance plays in healthy aging, this differential approach to maintain NAD^+^/NADH balance is a topic that warrants further research.

In conclusion, execution of the hepatic ISR by dietary SAAR corresponds with dynamic changes in the synthesis rates of individual liver proteins in male mice. Investigation of the kinetic proteome in the liver reveals four major signatures: first, a reduction in central metabolic pathways and processes relating to biotransformation and use of amino acids for energy; second, an increase in synthesis rates of proteins involved in the transport, preparation, and oxidation of fatty acids for energy; third, activation of the oxidative stress response and changes in NAD^+^/NADH-related tryptophan metabolism to support cellular redox balance; and finally, a dynamic restructuring of the ribo-interactome, whereby both RPs and RAPs are increased or decreased to adjust protein synthesis capacity and improve proteostasis maintenance. Interestingly, these signatures reflect overlap with other forms of dietary restriction and the adaptive response to starvation. These changes in the transcriptome and proteome, remarkably agnostic to dietary fat intake, work together to safely manage the increased fatty acid and energy flux, while supporting a slow growth phenotype that is synonymous with improved healthspan and extended lifespan. Future studies in females are needed to understand the basis for sex differences in the effect of dietary SAAR on liver proteostasis [11].

### Supplementary information

ESM 1: **Figure S1** Related to Figure 1. (A) Start and end body weights in grams for each experimental cohort (1, 3, 7, 14, 21 or 35 days on experimental diets, as indicated by grey headers), with groups being regular-fat control (RF.Ctrl), RF sulfur amino acid restricted (RF.SAAR), high-fat Ctrl (HF.Ctrl) and HF.SAAR. Dots connected by lines represent start and end body weights for individual animals in each cohort. (B) Normalized cumulative food intake in grams per gram body weight for each experimental cohort. (C) Daily normalized food intake (as energy). (D) Correlation between daily food intake (as energy) and serum levels of FGF21, with Pearson’s correlation coefficient (Pearson’s r or R) displayed for each timepoint. (E) Daily water intake. (F) Correlation between daily water intake and serum levels of FGF21, with Pearson’s r displayed for each timepoint. Data is displayed as mean ± standard error of the mean, with individual data points displayed as dots (n = 3-5 male mice per group). Within each timepoint (indicated in grey headers), bars without shared letters were statistically different at α = 0.05, as determined by 3-way ANOVA followed by pair-wise t-tests with (B, C and E) FDR correction for multiple comparisons (PDF 78.7 KB)

ESM 2: **Figure S2** Related to Figure 2. (A-D) Hepatic ratios and representative blots of (A) phosphorylated (at T899) GCN2 over total GCN2, (B) phosphorylated (at S51) eIF2α over total eIF2α, (C) phosphorylated (at T389) p70 S6K over total p70 S6K, and (D) phosphorylated (at S235/236) S6 over S6 in mice fed either a regular-fat control (RF.Ctrl), RF sulfur amino acid restricted (RF.SAAR), high-fat Ctrl (HF.Ctrl) or HF.SAAR diet for 35 days. Data is displayed as mean ± standard error of the mean, with individual data points displayed as dots (n = 3 male mice group). Displayed statistically significant (at α = 0.05) main effects were determined by 2-way ANOVA, and with “n.s.” indicating that no statistically significant differences were observed (PDF 94.5 KB)

ESM 3: **Figure S3** Related to Figure 3. (A) Differentially expressed gene (DEG, defined as q <0.05)-related volcano plot highlighting up and down regulated transcripts and total counts of each (inset bar graph) for high-fat sulfur amino acid restricted (HF.SAAR) vs. regular-fat (RF) SAAR (RF.SAAR), with integrated stress response (ISR)-related transcripts labelled if found among DEGs. (B) Gene set enrichment analysis-derived top 10 up and down (when applicable) regulated pathways based on gene set enrichment analysis of DEGs in HF.SAAR vs. RF.SAAR comparison. (C) Detailed lists of down DEGs comprising the gene ontology (GO) molecular function term “oxidoreductase activity” in each indicated comparison (left- or right-most columns), or both (middle columns) (PDF 80.5 KB)

ESM 4: **Figure S4** Related to Figure 4. (A) Top 5 WikiPathway (WP) terms for protein synthesis rate ratios increased (top) or decreased (bottom) in HF.SAAR vs. RF.SAAR. (B) Top 5 WikiPathway (WP) terms for protein synthesis rate ratios increased (top) or decreased (bottom) in HF.Ctrl vs. RF.Ctrl. Enrichment strength is indicated by shape size and false discovery rate (FDR) is displayed on the x-axis (PDF 190 KB)

ESM 5: **Figure S5** Related to Figure 5. (A) Hepatic expression of transcripts encoding core ribosomal proteins, expressed as fold change for the displayed comparisons, in mice fed a regular-fat control (RF.Ctrl) vs. RF sulfur amino acid restricted (RF.SAAR) and mice fed high-fat Ctrl (HF.Ctrl) vs. HF SAAR for seven days. Dots represent individual transcripts, with black dots signifying transcripts with FDR-adjusted p-values (padj) <0.05 and grey dots signifying padj >0.05 in both comparisons (PDF 52.7 KB)

ESM 6: **Figure S6** Whole liver lysate NAD+/NADH ratios are maintained during dietary sulfur amino acid restriction. (A) List of proteins with increased or decreased synthesis rate ratios falling into the WikiPathway term “Trp [tryptophan] metabolism” (WP79) in either the regular-fat control (RF.Ctrl) vs. RF sulfur amino acid restricted (RF.SAAR) comparison or the high-fat Ctrl (HF.Ctrl) vs. HF SAAR comparison. Underscored proteins signify ones found in both comparisons. (B) Distribution of synthesis rate ratios of observed aldehyde dehydrogenases (ADH5, ALDH1A1, ALDH1A2, ALDH1A3, ALDH1A7, ALDH1B1, ALDH1L1, ALDH1L2, ALDH2, ALDH3A1, ALDH3A2, ALDH3B1, ALDH3B2, ALDH3B3, ALDH4A1, ALDH7A1 and/or ALDH9A1) in the respective displayed comparisons. (C) Visualization of where KYNU and HAAO are situated in the de novo NAD+ biosynthesis pathway. (D) and (E) Hepatic NAD+/NADH ratios in the indicated groups after (D) seven or (E) 35 days of feeding experimental diets. (F) and (G) Hepatic ratios and representative blots of (F) PARP1 over total protein and (G) SIRT1 over total protein in mice fed either a RF.Ctrl (RF, or R and Ctrl, or C), RF.SAAR (RF, or R and SAAR, or S), HF.Ctrl (HF, or H and Ctrl, or C), or HF.SAAR (HF, or H and SAAR, or S) diet for 35 days. Data (in D, E, F and G) is displayed as mean ± standard error of the mean, with individual data points displayed as dots (n = 3 male mice per group). Statistically significant (at α = 0.05) differences were determined by 2-way ANOVA, with “n.s.” indicating that no statistically significant differences were observed (PDF 150 KB)

ESM 7: **Table S1** List of antibodies (XLSX 24.1 KB)

ESM 8: **Table S2** List of primers (XLSX 23.0 KB)

ESM 9: **Table S3** List of select differentially expressed genes. Comparisons are indicated by the respective tabs in the file (XLSX 27.9 MB)

ESM 10: **Table S4** List of integrated stress response-associated transcripts and proteins. Names are indicated in the column headers for protein names, gene names and UniProt IDs (XLSX 17.6 KB)

ESM 11: **Table S5** List of select hepatic kinetic proteomics data. Groups (regular-fat, control diet = RFC; regular-fat, SAAR diet = RFS; high-fat, control diet = HFC; high-fat, SAAR diet = HFS) are indicated by the respective tabs in the file (XLSX 214 KB)

ESM 12: **Table S6** Unique proteins identified using kinetic proteomics. List of select kinetic proteomics data for unique lists of proteins found in only one group (XLSX 24.9 KB)

ESM 13: **Table S7** Pathway enrichment data. List of WikiPathway (WP) terms associated to the respective comparisons. Comparisons are indicated by the respective tabs in the file (XLSX 24.8 KB)

ESM 14: **Table S8** Day seven proteomics data. List of select comparisons of hepatic proteomics data from day seven (XLSX 136 KB)

ESM 15: **Table S9** Select synthesis rate ratios of identified ribosome-associated proteins. List of select identified ribosome-associated proteins (RAPs) in indicated comparisons. Comparisons are indicated by the respective tabs in the file (XLSX 68.7 KB)

ESM 16: **Jonsson Supplement Table Legend** (DOCX 13.9 KB)

ESM 17: **Jonsson Supplement Figure Legend** (DOCX 18.3 KB)

ESM 18: **Figure S7** (EPS 2.64 MB)

ESM 19: **Figure S8** (EPS 1.81 MB)

ESM 20: **Figure S9** (EPS 2.63 MB)

ESM 21: **Figure S10** (EPS 693 KB)

ESM 22: **Figure S11** (EPS 8.33 MB)
