# Supplementary material for: Kinetic proteomics identifies targeted changes in liver metabolism and the ribo-interactome by dietary sulfur amino acid restriction
Source: GeroScience. 2023 Mar 28;45(4):2425–41. doi: 10.1007/s11357-023-00758-w (PMC10651627; doi:10.1007/s11357-023-00758-w)
Supplement: Supplementary file 6 — Figure S6 Whole liver lysate NAD+/NADH ratios are maintained during dietary sulfur amino acid restriction. (A) List of proteins with increased or decreased synthesis rate ratios falling into the WikiPathway term “Trp [tryptophan] metabolism” (WP79) in either the regular-fat control (RF.Ctrl) vs. RF sulfur amino acid restricted (RF.SAAR) comparison or the high-fat Ctrl (HF.Ctrl) vs. HF SAAR comparison. Underscored proteins signify ones found in both comparisons. (B) Distribution of synthesis rate ratios of observed aldehyde dehydrogenases (ADH5, ALDH1A1, ALDH1A2, ALDH1A3, ALDH1A7, ALDH1B1, ALDH1L1, ALDH1L2, ALDH2, ALDH3A1, ALDH3A2, ALDH3B1, ALDH3B2, ALDH3B3, ALDH4A1, ALDH7A1 and/or ALDH9A1) in the respective displayed comparisons. (C) Visualization of where KYNU and HAAO are situated in the de novo NAD+ biosynthesis pathway. (D) and (E) Hepatic NAD+/NADH ratios in the indicated groups after (D) seven or (E) 35 days of feeding experimental diets. (F) and (G) Hepatic ratios and representative blots of (F) PARP1 over total protein and (G) SIRT1 over total protein in mice fed either a RF.Ctrl (RF, or R and Ctrl, or C), RF.SAAR (RF, or R and SAAR, or S), HF.Ctrl (HF, or H and Ctrl, or C), or HF.SAAR (HF, or H and SAAR, or S) diet for 35 days. Data (in D, E, F and G) is displayed as mean ± standard error of the mean, with individual data points displayed as dots (n = 3 male mice per group). Statistically significant (at α = 0.05) differences were determined by 2-way ANOVA, with “n.s.” indicating that no statistically significant differences were observed (PDF 150 KB) [file 11357_2023_758_MOESM6_ESM.pdf]

Figure S6.

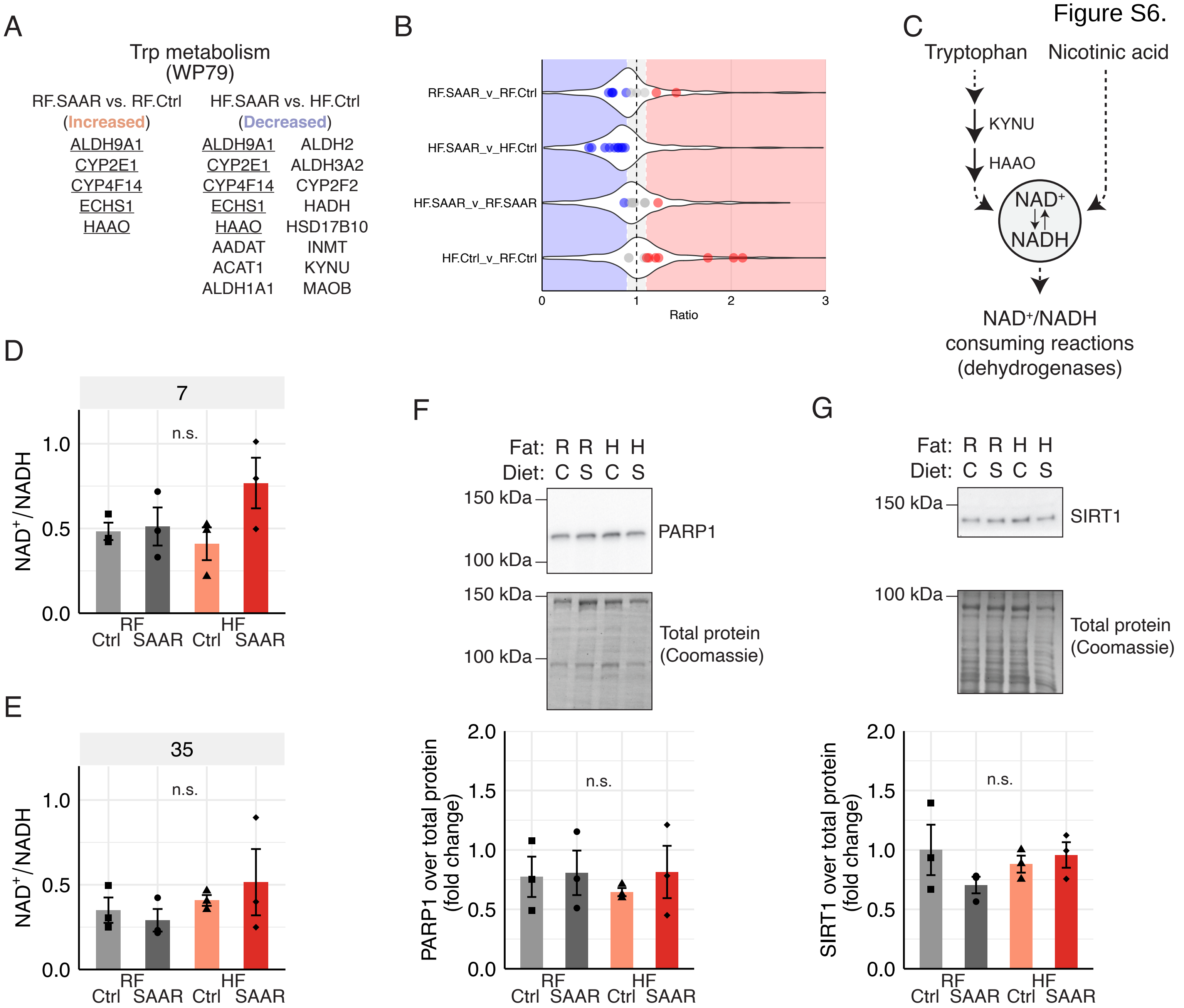

A
Trp metabolism (WP79)
RF.SAAR vs. RF.Ctrl (Increased)
ALDH9A1
CYP2E1
CYP4F14
ECHS1
HAAO
HF.SAAR vs. HF.Ctrl (Decreased)
ALDH9A1 ALDH2
CYP2E1 ALDH3A2
CYP4F14 CYP2F2
ECHS1 HADH
HAAO HSD17B10
AADAT INMT
ACAT1 KYNU
ALDH1A1 MAOB
B
RF.SAAR_v_RF.Ctrl
HF.SAAR_v_HF.Ctrl
HF.SAAR_v_RF.SAAR
HF.Ctrl_v_RF.Ctrl
0 1 2 3
Ratio
C
Tryptophan
Nicotinic acid
KYNU
HAAO
NAD+
NADH
NAD+/NADH consuming reactions (dehydrogenases)
D
7
NAD+/NADH
n.s.
RF HF
Ctrl SAAR Ctrl SAAR
E
35
NAD+/NADH
n.s.
RF HF
Ctrl SAAR Ctrl SAAR
F
Fat: R R H H
Diet: C S C S
150 kDa
100 kDa
PARP1
Total protein (Coomassie)
PARP1 over total protein (fold change)
n.s.
RF HF
Ctrl SAAR Ctrl SAAR
G
Fat: R R H H
Diet: C S C S
150 kDa
SIRT1
100 kDa
Total protein (Coomassie)
SIRT1 over total protein (fold change)
n.s.
RF HF
Ctrl SAAR Ctrl SAAR
