# Supplementary material for: Kinetic proteomics identifies targeted changes in liver metabolism and the ribo-interactome by dietary sulfur amino acid restriction
Source: GeroScience. 2023 Mar 28;45(4):2425–41. doi: 10.1007/s11357-023-00758-w (PMC10651627; doi:10.1007/s11357-023-00758-w)
Supplement: Supplementary file 16 — Jonsson Supplement Table Legend (DOCX 13.9 KB) [file 11357_2023_758_MOESM16_ESM.docx]

Supplementary Table Captions

**Table S1. List of antibodies.**

**Table S2. List of primers.**

**Table S3. List of select differentially expressed genes**. Comparisons are indicated by the respective tabs in the file.

**Table S4.** **List of integrated stress response-associated transcripts and proteins.** Names are indicated in the column headers for protein names, gene names and UniProt IDs.

**Table S5.** **List of select hepatic kinetic proteomics data.** Groups (regular-fat, control diet = RFC; regular-fat, SAAR diet = RFS; high-fat, control diet = HFC; high-fat, SAAR diet = HFS) are indicated by the respective tabs in the file.

**Table S6. Unique proteins identified using kinetic proteomics.** List of select kinetic proteomics data for unique lists of proteins found in only one group.

**Table S7. Pathway enrichment data.** List of WikiPathway (WP) terms associated to the respective comparisons. Comparisons are indicated by the respective tabs in the file.

**Table S8. Day seven proteomics data.** List of select comparisons of hepatic proteomics data from day seven.

**Table S9. Select synthesis rate ratios of identified ribosome-associated proteins.** List of select identified ribosome-associated proteins (RAPs) in indicated comparisons. Comparisons are indicated by the respective tabs in the file.
