# Supplementary material for: Kinetic proteomics identifies targeted changes in liver metabolism and the ribo-interactome by dietary sulfur amino acid restriction
Source: GeroScience. 2023 Mar 28;45(4):2425–41. doi: 10.1007/s11357-023-00758-w (PMC10651627; doi:10.1007/s11357-023-00758-w)
Supplement: Supplementary file 17 — Jonsson Supplement Figure Legend (DOCX 18.3 KB) [file 11357_2023_758_MOESM17_ESM.docx]

Supplementary Material

Supplementary Figure Captions

**Figure S1. Related to Figure 1.** (A) Start and end body weights in grams for each experimental cohort (1, 3, 7, 14, 21 or 35 days on experimental diets, as indicated by grey headers), with groups being regular-fat control (RF.Ctrl), RF sulfur amino acid restricted (RF.SAAR), high-fat Ctrl (HF.Ctrl) and HF.SAAR. Dots connected by lines represent start and end body weights for individual animals in each cohort. (B) Normalized cumulative food intake in grams per gram body weight for each experimental cohort. (C) Daily normalized food intake (as energy). (D) Correlation between daily food intake (as energy) and serum levels of FGF21, with Pearson’s correlation coefficient (Pearson’s r or R) displayed for each timepoint. (E) Daily water intake. (F) Correlation between daily water intake and serum levels of FGF21, with Pearson’s r displayed for each timepoint. Data is displayed as mean ± standard error of the mean, with individual data points displayed as dots (n = 3-5 male mice per group). Within each timepoint (indicated in grey headers), bars without shared letters were statistically different at α = 0.05, as determined by 3-way ANOVA followed by pair-wise t-tests with (B, C and E) FDR correction for multiple comparisons.

**Figure S2. Related to Figure 2.** (A-D) Hepatic ratios and representative blots of (A) phosphorylated (at T899) GCN2 over total GCN2, (B) phosphorylated (at S51) eIF2α over total eIF2α, (C) phosphorylated (at T389) p70 S6K over total p70 S6K, and (D) phosphorylated (at S235/236) S6 over S6 in mice fed either a regular-fat control (RF.Ctrl), RF sulfur amino acid restricted (RF.SAAR), high-fat Ctrl (HF.Ctrl) or HF.SAAR diet for 35 days. Data is displayed as mean ± standard error of the mean, with individual data points displayed as dots (n = 3 male mice group). Displayed statistically significant (at α = 0.05) main effects were determined by 2-way ANOVA, and with “n.s.” indicating that no statistically significant differences were observed.

**Figure S3. Related to Figure 3.** (A) Differentially expressed gene (DEG, defined as q <0.05)-related volcano plot highlighting up and down regulated transcripts and total counts of each (inset bar graph) for high-fat sulfur amino acid restricted (HF.SAAR) vs. regular-fat (RF) SAAR (RF.SAAR), with integrated stress response (ISR)-related transcripts labelled if found among DEGs. (B) Gene set enrichment analysis-derived top 10 up and down (when applicable) regulated pathways based on gene set enrichment analysis of DEGs in HF.SAAR vs. RF.SAAR comparison. (C) Detailed lists of down DEGs comprising the gene ontology (GO) molecular function term “oxidoreductase activity” in each indicated comparison (left- or right-most columns), or both (middle columns).

**Figure S4. Related to Figure 4.** (A) Top 5 WikiPathway (WP) terms for protein synthesis rate ratios increased (top) or decreased (bottom) in HF.SAAR vs. RF.SAAR. (B) Top 5 WikiPathway (WP) terms for protein synthesis rate ratios increased (top) or decreased (bottom) in HF.Ctrl vs. RF.Ctrl. Enrichment strength is indicated by shape size and false discovery rate (FDR) is displayed on the x-axis.

**Figure S5. Related to Figure 5.** (A) Hepatic expression of transcripts encoding core ribosomal proteins, expressed as fold change for the displayed comparisons, in mice fed a regular-fat control (RF.Ctrl) vs. RF sulfur amino acid restricted (RF.SAAR) and mice fed high-fat Ctrl (HF.Ctrl) vs. HF SAAR for seven days. Dots represent individual transcripts, with black dots signifying transcripts with FDR-adjusted p-values (padj) <0.05 and grey dots signifying padj >0.05 in both comparisons.

**Figure S6. Whole liver lysate NAD+/NADH ratios are maintained during dietary sulfur amino acid restriction.** (A) List of proteins with increased or decreased synthesis rate ratios falling into the WikiPathway term “Trp [tryptophan] metabolism” (WP79) in either the regular-fat control (RF.Ctrl) vs. RF sulfur amino acid restricted (RF.SAAR) comparison or the high-fat Ctrl (HF.Ctrl) vs. HF SAAR comparison. Underscored proteins signify ones found in both comparisons. (B) Distribution of synthesis rate ratios of observed aldehyde dehydrogenases (ADH5, ALDH1A1, ALDH1A2, ALDH1A3, ALDH1A7, ALDH1B1, ALDH1L1, ALDH1L2, ALDH2, ALDH3A1, ALDH3A2, ALDH3B1, ALDH3B2, ALDH3B3, ALDH4A1, ALDH7A1 and/or ALDH9A1) in the respective displayed comparisons. (C) Visualization of where KYNU and HAAO are situated in the *de novo* NAD^+^ biosynthesis pathway. (D) and (E) Hepatic NAD^+^/NADH ratios in the indicated groups after (D) seven or (E) 35 days of feeding experimental diets. (F) and (G) Hepatic ratios and representative blots of (F) PARP1 over total protein and (G) SIRT1 over total protein in mice fed either a RF.Ctrl (RF, or R and Ctrl, or C), RF.SAAR (RF, or R and SAAR, or S), HF.Ctrl (HF, or H and Ctrl, or C), or HF.SAAR (HF, or H and SAAR, or S) diet for 35 days. Data (in D, E, F and G) is displayed as mean ± standard error of the mean, with individual data points displayed as dots (n = 3 male mice per group). Statistically significant (at α = 0.05) differences were determined by 2-way ANOVA, with “n.s.” indicating that no statistically significant differences were observed.
